# Comparative cyto-histological study of needle tip aspirates and entry sites after intravitreal injection using different needle types

**DOI:** 10.1371/journal.pone.0174467

**Published:** 2017-07-10

**Authors:** Lyubomyr Lytvynchuk, Andrij Sergienko, Iryna Savytska, Réka Albert, Carl Glittenberg, Susanne Binder, Goran Petrovski

**Affiliations:** 1 Department of Ophthalmology, Justus-Liebig-University Giessen, Eye Clinic, University Hospital Giessen and Marburg GmbH, Campus Giessen, Giessen, Germany; 2 Professor Sergienko Eye Clinic, Vinnycia, Ukraine; 3 Karl Landsteiner Institute for Retinal Research and Imaging, Vienna, Austria; 4 Stem Cells and Eye Research Laboratory, Department of Ophthalmology, Faculty of Medicine, Albert Szent-Gyorgyi Clinical Center, University of Szeged, Szeged, Hungary; 5 Department of Experimental Surgery, A.A.Shalimov National Institute of Surgery and Transplantology, National Academy of Medical Science of Ukraine, Kyiv, Ukraine; 6 Department of Ophthalmology, Rudolf Foundation Clinic, Vienna, Austria; 7 Center for Eye Research, Department of Ophthalmology, Oslo University Hospital and University of Oslo, Oslo, Norway; Wayne State University School of Medicine, UNITED STATES

## Abstract

A comparison of the cellular content of needle tip aspirates and entry sites after transconjunctival intravitreal injection (IVI) using different needle types was performed. White outbred rats and human cadaver eyes were used for IVI by hypodermic 27 gauge (G) and 30G needles, and spinal anesthesia Pencan 27G needles. Aspiration of vitreous for quantitative morphological and cell cultivation analysis, as well as cyto-histological analysis of aspirates and entry sites were carried out. The most common cells in the aspirates from all needle types were conjunctival epithelial-, ciliary body non-pigmented epithelial- and sclerocyte-like cells and granular proteins. Crystallized vitreous specimens were present in each aspirate. The entry sites of hypodermic needles showed marked trauma in all wall layers of rat and human eyes accompanied by cellular destruction and hemorrhages. Pencan 27G needle caused less tissue trauma with partial reposition of sclerocytes. Transconjunctival IVIs with hypodermic 27G and 30G, and Pencan 27G needles result in trauma of all layers of the eyeball. The possible consequences of cellular content being cut and injected into the eye, as well as the entry site wound shape deserve future consideration and improvements.

## Introduction

During the last decade, the number of intravitreal injections (IVI) of different drugs performed throughout the world has significantly increased[1–4]. The outcome of multiple studies reveals both therapeutic effectiveness and risks of complications after intravitreal drug delivery[1,5]. Endophthalmitis is among the most severe and sight threatening complications after IVIs, ranging from 0.018% to 1.4% of the cases, in spite of having good antibiotic prophylaxis[6–9]. The side effects of IVI have been non-infectious endophthalmitis as well[10,11]. The prophylactic use of antibiotics before and after IVIs does not prevent, and according to some studies increases the risk of endophthalmitis[10,12,13]. Despite a low complications rate, the influence of different IVI techniques and use of different needle types on the frequency of complications has not been well studied [14–16]. To our knowledge, all needle types that have been used for IVI (hypodermic or subcutaneous) have trephine-like configuration with outer and inner sharp edges of the tip[17]. This specific design facilitates cut of a column of all tissues that are penetrated by the needle. There is a very high risk this cellular column gets into the vitreous cavity during IVI, thus causing cellular contamination. Besides that, bacteria present for whatever reason in the conjunctiva, including such that are intracellular (e.g. *Chlamydia* species), can gain entrance into the vitreous cavity and be the cause of early or delayed reaction[18,19].

The vitreous body is a natural cultivation medium full of regulating factors that can either stimulate or inhibit proliferation of various cells[20]. Possible growth of the cells unintentionally injected into the eye creates a risk for complications associated with proliferation, and septic or aseptic (autoimmune) inflammation. The extent of the eye wall trauma during IVI is associated with pain and vitreous reflux[21,22]. Subconjuctival reflux of the vitreous or injected drug, which is often seen after IVI, is conditioned not only by the amount of injected drug, but also by the IVI technique, type of the needle and the vitreous state[21]. The technique of IVI and the needle size can be responsible for the risk of contamination. The configuration of the entry site after fine needle penetration and its self-sealing properties are important for preserving a closed system free or resistant to infection.

The present study uses the concept of fine needle biopsy as mainly used in oncology during biopsy taking[23]. With this technique, a fine hypodermic needle is introduced into the target tissue and a certain amount of cellular content is aspirated into the syringe. Consequently, a cytological study or cultivation of aspirated material is performed. Additionally, transconjunctival IVIs were applied with a spinal anesthesia needle with a pencil-point tip, originally designed for better recognition of passing the dura mater and arachnoid in lumbar puncture, and then correlated to standard subcutaneous needles[24].

We hereby compare the cellular content of the needle tip aspirates after intravitreal penetration using three different types of needles in rats and human cadaver eyes using cyto-histological analysis, and evaluate the ex vivo cultivated material as well as compare the entry sites of different needle types being used.

## Methods

### Ethics statement

All experiments performed complied to the Guidelines of the Helsinki Declaration and the ARVO statement for use of animals in ophthalmic and visual research, and were approved by the Regional and Institutional Research Ethics Committee (IREC) at the University of Debrecen, Hungary (183/2013.(14415/2013/EKU)). Eyes were enucleated from a cadaver and used only after being checked in the national register for transplantation for being compliant to the presumed consent rule and the Cornea Bank experts found them not suitable for corneal transplantation, thus permitting use in research under the above ethical approval. The experimental animal study was approved by the Ethical Committee according to the rules of “Scientific and practical recommendations for hosting the animals and experimental work” Ministry of Health Care (Protocol #5, from 19.06.2002) and The Law of Ukraine about defending the animals from violence (No 1759-VI from 15.12.2009).

### Needles

**Fig 1:**
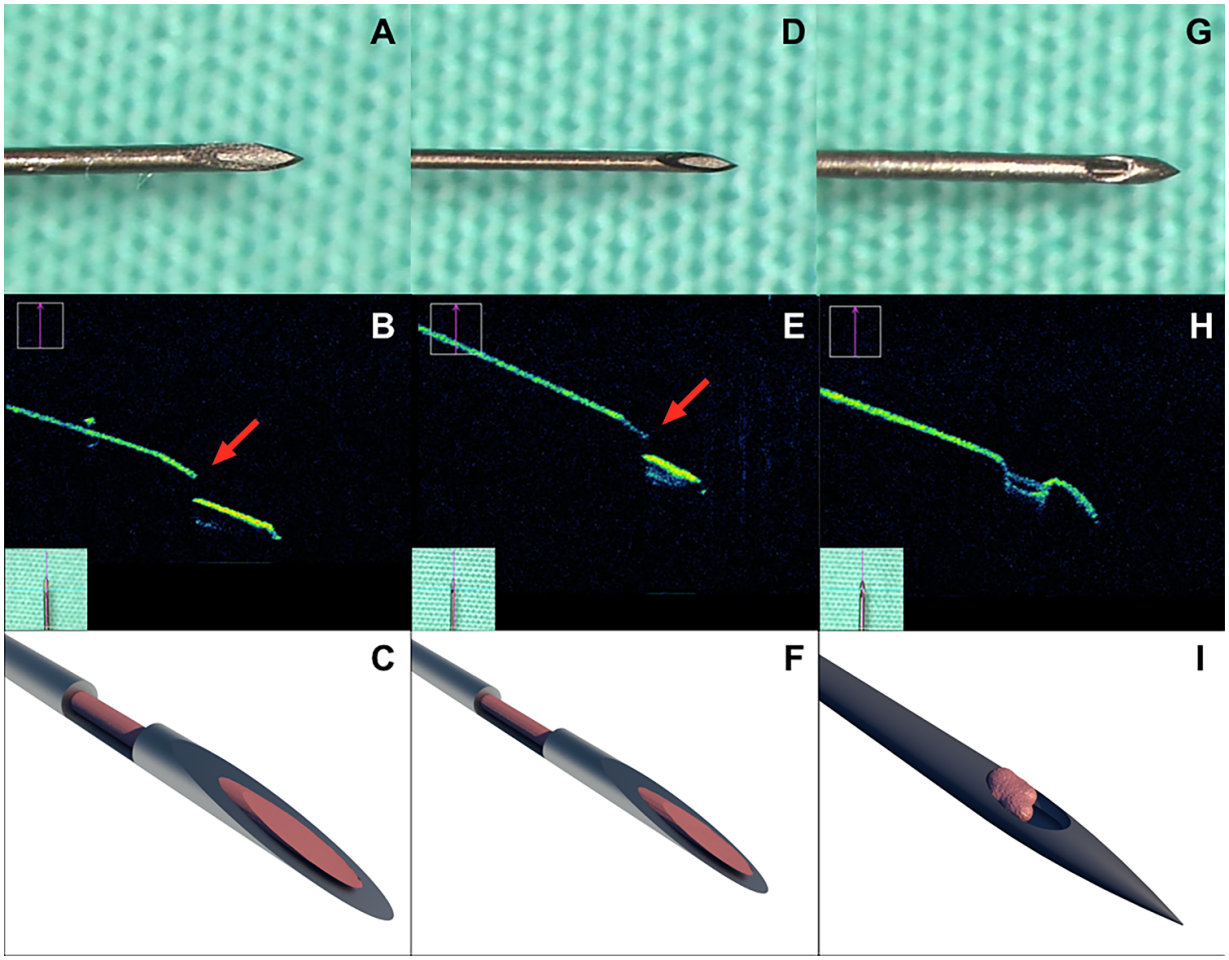
Enlarged view of needles used in experiments. Enlarged view of the hypodermic 27G (a), 30G (d) needles and Pencan 27G (g) needle. iSD-OCT of the hypodermic 27G (b), 30G (e) needles and Pencan 27G (h) needle. Red arrows indicate the sharp inner edge of the tip of the hypodermic needles (b, e). Schematic view of the hypodermic 27G (c), 30G (f) needles and Pencan 27G (i) needle with cellular content inside the needle’s tips (pink).

**Fig 2:**
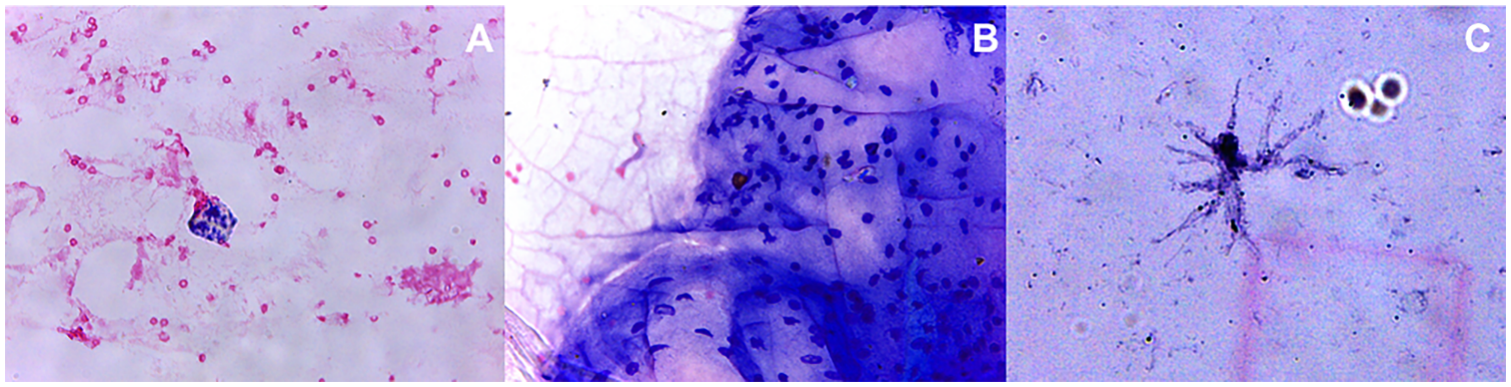
Aspirates taken by different needle types applied to rat eyes. Conjunctival epithelial- (a), ciliary body epithelial- (b) and sclerocyte-like cells (c) found in aspirates taken by different needles applied to rat eyes.

Three types of needles were used in this study: 1) standard hypodermic 27 Gauge (G) needle (BD, Franklin Lakes, New Jersey, USA) with outer diameter of 0.41 mm, inner diameter of 0.21 mm, wall of 0.101 mm, and length of 13 mm (Fig 1a, 1b and 1c); 2) standard hypodermic 30G needle that is regularly used for IVI (BD, Franklin Lakes, New Jersey, USA) with outer diameter of 0.31 mm, inner diameter of 0.16 mm, wall of 0.076 mm, bevel degree 12° (A-bevel), and length of 13 mm (Fig 1d, 1e and 1f). The 27G and 30G hypodermic needles have a trephin-like design of the tip with sharp inner and outer edges due to polishing of the tubes during the manufacturing[17]. The needles are made of stainless steel; 3) pencil-point needle for spinal anesthesia Pencan 27G (B.Braun, Melsungen, Germany) with outer diameter of 0.42 mm, inner diameter of 0.16 mm, wall of 0.076 mm, and length of 88mm[25]. The latter needle type has a pic-like design of the tip with a side port, which margins are blunt (Fig 1g, 1h and 1i). It was designed for safe single shot handling of the spinal anesthesia and diagnostic lumbar puncture that is facilitated by easy identification of passing the dura mater and arachnoid. The needle has a pencil-point atraumatic tip design which protects against vascular puncture and nerve damage. It is reported by the manufacturer that there is no tip deformation even after bone contact.

The 1.0cc syringes were pre-loaded with 0.02cc of balanced salt solution (BSS) and connected to each needle in order to dissolve and flush out aspirated cells. Every needle was used only once.

Intraoperative spectral domain optical coherence tomography (iSD-OCT) with Rescan 700 (Zeiss, Oberkochen, Germany) was used to image and compare the tips of the needles. The iSD-OCT technical data were the following: volume scans 512 x 128 cube, scan length of 6 mm: 512x128 cube, wavelength: 840 nm, iSD-OCT scans with length 6 mm, scan mode cross-hair, scanning speed: 27.000 A-scans per second, refresh rate: 5 Hz to 50 Hz, axial resolution: 5.5 μm in tissue. The iSD-OCT images were post-processed and analyzed. Based on the photographic and iSD-OCT images, the schematic drawing of the needle tips was created using graphic design software (Cinema 4D, MAXON Computer GmbH, Friedrichsdorf, Germany) to visualize the cellular content cut and captured by the needle tips (Fig 1c, 1f and 1e).

### Intravitreal penetration and aspiration performed on rat and human cadaver eyes

The animal study was conducted on 30 eyes of 30 Wistar white outbred albino rats (age: 6 months, weight: 600–800 g). Anesthesia was facilitated by subcutaneous injection of 0.2mL of 1% thiopental solution and 0.4mL of 20% oxybutirate-Na solution. Ten IVIs were performed with each hypodermic 27G, 30G and spinal anesthesia Pencan 27G needles. Penetration of the needles through the ciliary body 1 mm posterior to the limbus area in oblique fashion and directed toward central retina under the control of operating microscope was carried out to avoid lens and peripheral retina trauma[26,27]. Each injection was performed on the right eye of the animal.

The human study was conducted on two eyes of a 77year-old female cadaver enucleated within 12hrs from death. Only hypodermic 30G and spinal anesthesia Pencan 27G needles were used. Ten IVIs were performed with each needle type. Transconjunctival intravitreal penetration with the needles into the vitreous cavity was performed perpendicularly to the sclera, 4 mm posterior to the limbus.

Instead of injection, aspiration of 0.01cc of vitreous body was performed. The procedure was carried out under an operating microscope in order to control the position of the needle tip and needle orifice in the narrow vitreous cavity of the rat’s eye. To perform IVIs with Pencan27G needles that have the length of 88 mm, the surgeon used both hands while performing the penetration, avoiding bending of the needle.

The target angle for all IVIs relatively to sclera was 90 degrees. The corneas of the rats and cadaver eyes were marked with a linear abrasion in the meridian that corresponded to the needle's entry site for easy localization of the penetration place and adequate preparation of the paraffin blocks and sections[27]. In animal experiments, the euthanasia was performed at the same day after the IVI by an overdose of a 10% sodium thiopental solution.

### Cytological study of needle tip aspirates

Aspirated material containing pre-loaded BSS was evacuated through the same IVI needle tip and onto glass slides, where it was fixed with 4% formalin and stained with azure-2-eosin. The number of each cell type was assessed in 30 equal visual fields in the shape of equal squares of 160 μm^2^. The cells were recognized by their morphological characteristics, and their quantity counted in each square of all cases studied. The cytological analysis was performed with the use of light microscope HD Microscope Camera CC50HD (Leica MikrosystemeVertrieb GmbH, Wetzlar, Germany) using magnification of 100X and 400X.

The study aimed to reveal and recognize the cells of ocular tissues through which the needles penetrated (conjunctiva, sclera, ciliary body, vitreous). Separate cells were counted and presented in numbers, and the cellular complexes (granular proteins, conjunctival cell conglomerates) were estimated by their area of distribution in 1 mm^2^. The granulated basophilic protein sediments were considered to be a result of cellular damage. Conjunctival cell complexes were the group of so called fasciated conjunctival cells, which connected with each other by intracellular junctions and were supported by a basal membrane.

### Histological study of needle entry sites in tissues from rat and human cadaver eyes

All rats were euthanized within 1 hour after the intravitreal injection. After euthanasia of the rats, the right eyes were enucleated, and the corneo-scleral segments that corresponded to the IVI were excised and fixed in 10% formalin solution. Similar excision of the previously marked corneo-scleral segments was performed on the cadaver eyes. Consequent washing and embedding of the prepared tissue samples into paraffin was used by applying a combined method with intermediate medium of 2% celloidin solution in alcohol-ether mixed at a ratio of 1:1. Slices 8 μm thick were stained with azure-2-eosin and hematoxyllin and eosin [23]. The right eye sections in rats were compared to the left eye sections considered to be controls. The histological analysis was performed with the use of light microscope (Leica ICC50HD, Germany, magnification: 100X and 400X).

### Cellular cultivation study

Aspirated material of the cadaver eyes (n = 5 per needle type per eye) were washed and seeded into 96 well plates containing medium consisting of Dulbecco-modified Eagle’s medium nutrient mixture—F12 HAM (DMEM-F12, Sigma-Aldrich, St. Louis, MO, USA), supplemented with 20% fetal calf serum (FCS) (Gibco; Gibco, London, UK), 200 mM/mL L-glutamine (Sigma-Aldrich, St. Louis, MO, USA), 10,000 U/mL penicillin-10 mg/mL streptomycin (Sigma-Aldrich, St. Louis, MO, USA); 24hrs after seeding, the adherent cells were cultivated in total of 200 μL medium. Feeding of the cells occurred on each alternate day. The growth of the cells was monitored under phase contrast microscope regularly. After 4 weeks of cultivation, the cells were fixed in 4% formalin and the nuclei stained by Hoechst (1:800, 10 min). The histological analysis was performed using an EVOS FL Cell Imaging System (Thermo Fisher Scientific, Waltham, MA, USA, magnification: 40X and 100X).

### Statistical analysis

The study results were analyzed statistically using the software STATISTICA 10 (StatSoft, Inc., OK, USA) according to the following methods: descriptive statistics, frequency tables, correlation matrices and t-test for Independent Samples. A p-value of <0.05 was considered statistically significant.

## Results

### Cytological study of needle tip aspirates

Cytological analysis of the needle tip aspirates using three needle types on rat and human cadaver eyes showed presence of cellular content in each case following transconjunctival penetration. The cellular material generally consisted of conjunctival-, scleral-, ciliary body- cells and vitreous body remnants (Fig 2). Conjunctival cellular conglomerates were found, such as cells connected to each other by intercellular junctions and supported by a basal membrane. Additionally, granular protein complexes were noticed that were present in all three different needle types being used. The cellular content showed presence of singular erythrocytes and lymphocytes as well.

Analysis of iSD-OCT showed the sharp inner edge of the hypodermic needle tips (Fig 1b and 1e, red arrows), andvisualised the blunt margins of the orifice of the Pencan 27G needle tip, as well as the pencil-like configuration of the tip (Fig 1h, pink).

#### Study on rat eyes

The absolute quantity and appearance of conjunctival epithelial cells in a representative sample of hypodermic 27G needle aspirates was 56, in case of hypodermic 30G needle—66, and in case of Pencan 27G needle—170 cells, respectively (Fig 2). Similarly, the absolute amount of ciliary body epithelial cells in a representative case of hypodermic 27G, 30G and Pencan 27G needle aspirates was 69, 7 and 136 cells, while that of sclerocytes was 24, 14 and 17 cells, respectively. There were also few (2) to none neuronal/retinal cells found in the hypodermic 27G and 30G needle aspirates, respectively, and more of that cell type (9) was seen in the Pencan 27G needle aspirates (Fig 2). The amount of conjunctival epithelial- and ciliary body epithelial- cells was significantly higher in the Pencan 27G needle aspirates compared to the hypodermic 27G and 30G (p = 0.05). The number of sclerocytes was higher in the hypodermic 27G needles compared to the hypodermic 30G and Pencan 27G needles, with no significant difference between the last two needle types.

**Fig 3:**
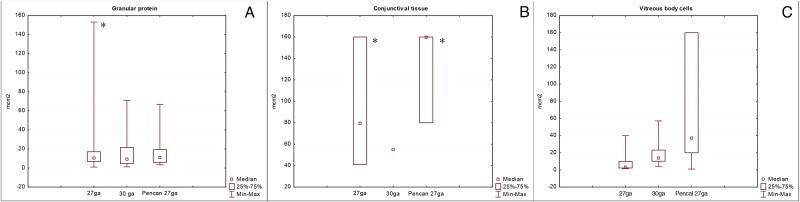
The amount of granular proteins (a), conjunctival cell complexes (b) and vitreous body cells (c) in the aspirates taken by different needle types applied to rat eyes. (*p<0.05).

In all 30 rat cases of IVI, the azure-2-eosin staining showed presence of basophilic granulated sediment that represents granular proteins aggregated by the methanol fixation (Fig 3). These proteins were likely vessel exudates, connective tissue and sclera as a consequence of the cellular damage caused by the needle. The amount of granulated sediment in the aspirates taken from the hypodermic 27G needles was significantly higher compared to the hypodermic 30G and Pencan 27G needles (p<0.05) (Fig 3a). At the same time, the amount of conjunctival cell complexes prevailed in the cases where hypodermic 27G and Pencan 27G needles were used. However, presence of basal membrane was seen more frequently in aspirates taken from hypodermic 27G and 30G needles, as compared to Pencan 27G needles (p<0.05) (Fig 3b). The single conjunctival epithelial cells were seen on the slides more often compared to conjunctival cell complexes. The quantity of crystallized vitreous remnants that were found in each aspirate is depicted on Fig 3c. The comparison between the different types of needles was not significant, since aspiration of the vitreous during the experiment was obligatory to ensure aspiration of the cellular cylinder being cut.

#### Study on human cadaver eyes

**Fig 4:**
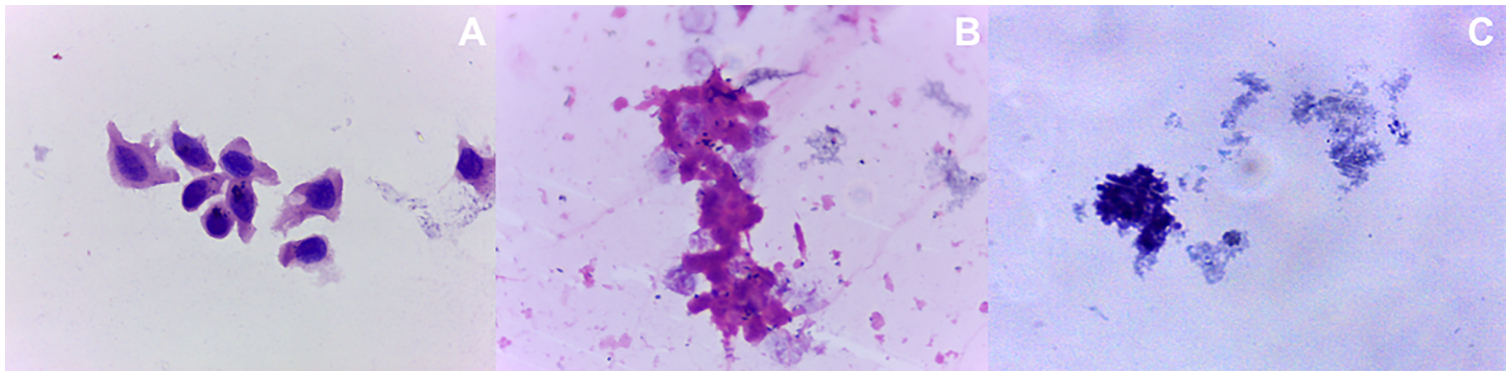
Aspirates taken by different needles applied to cadaver eyes. Conjunctival epithelial cells alone (a) and in complexes (b), and granular proteins(c) found in aspirates taken by different needles applied to cadaver eyes.

**Fig 5:**
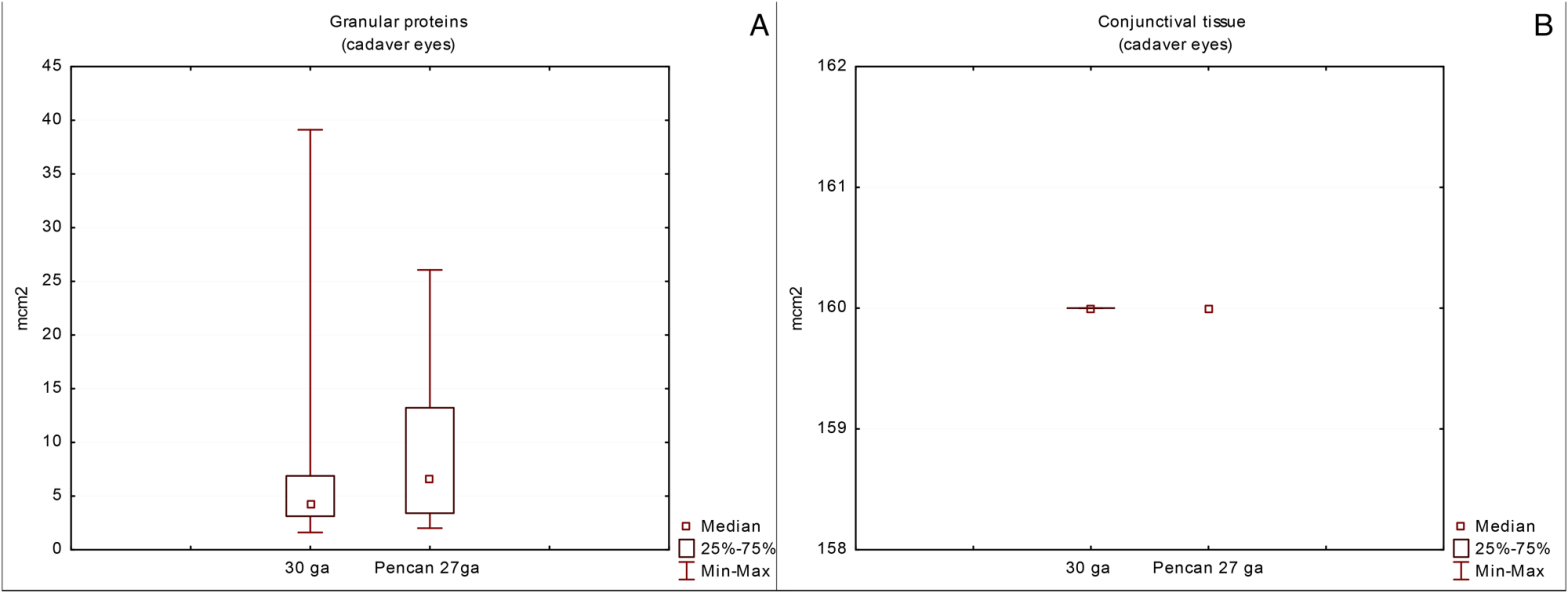
The amount of conjunctival cell complexes (a) and granular proteins (b) in aspirates taken by hypodermic 30G- and Pencan 27G- needles applied to cadaver eyes.

Cytological study of the needle tip aspirates in human cadaver eyes showed less cellular material (Fig 4). The absolute quantity and appearance of conjunctival cells in a representative sample of aspirates from hypodermic 30G and Pencan 27G needles was 42 and 95 cells, respectively. The majority of the conjunctival cells presented with a frothy cytoplasm and poor delineation of the nuclear margins (Fig 4a and 4b). The total square area of the granular protein complexes from hypodermic 30G needles was 328.58 μm^2^, while that from Pencan 27G needles was 483.32 μm^2^ (Figs 4 and 5a). The amount of conjunctival cell complexes found in aspirates from hypodermic 30G needles was 14, and from Pencan 27G needles it was 39 cells (Fig 5b). There were neither ciliary body epithelial cells nor sclerocytes or vitreous remnants noticed in the cadaver samples being tested from both needle types. The difference between the needles types regarding granular protein complexes and conjunctival cell complexes was not statistically significant.

### Histological study of needle entry sites

#### Study on rat eyes

**Fig 6:**
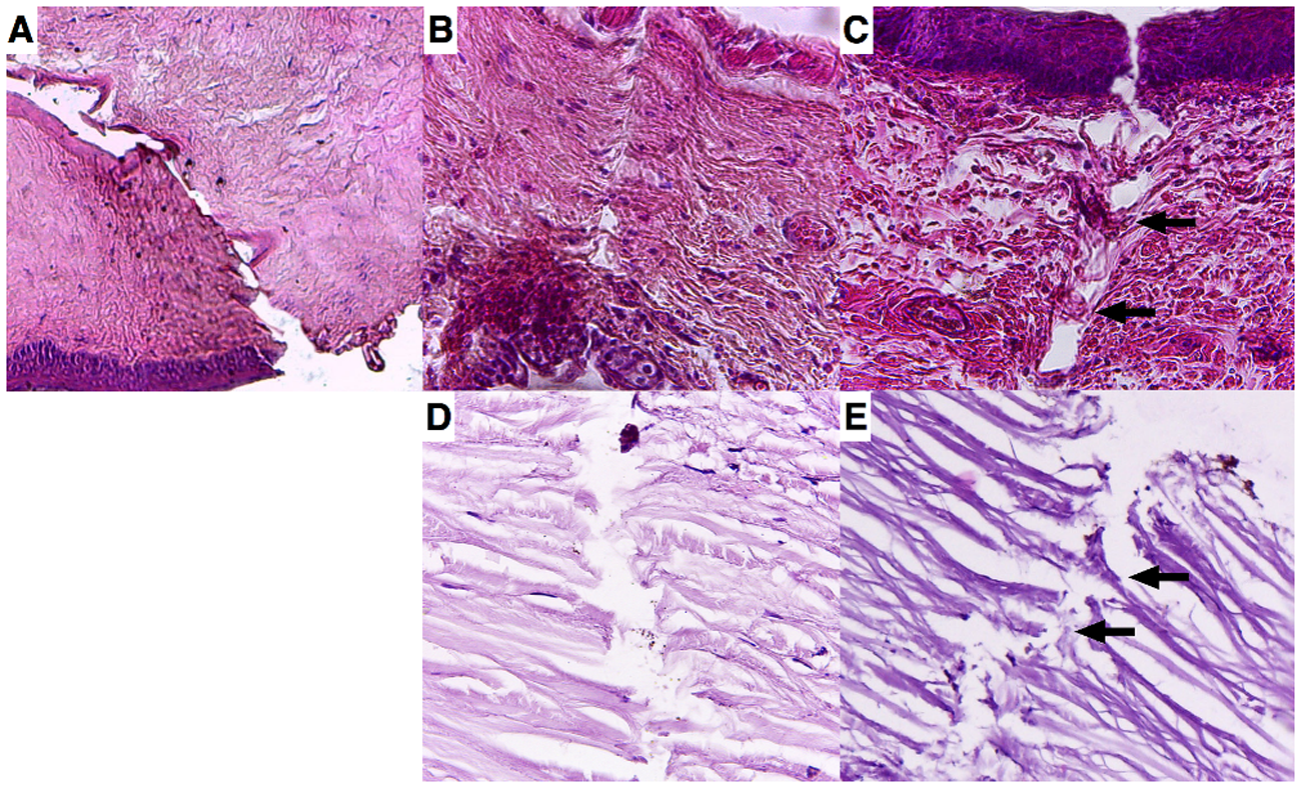
Histological cuts of the needle entry sites after intravitreal injection of rat and human eyes. Histological cuts of the needle entry sites after intravitreal injection (IVI) with hypodermic 27G and 30G needles (a, b) and Pencan 27G needle (c) on rat eyes. By analogy, histological cuts of the needle entry sites after IVI with hypodermic 30G needle (d) and Pencan 27G(e) on cadaver eyes is also shown. Black arrows indicate partial reposition of the collagen fibrils within the Pencan 27G needle track on rat eyes (c) and cadaver eyes (e).

Penetration of the needles through the ocular tissues caused damage of all structures of the eye wall that were surpassed on the way to the vitreous. In cases where IVI was performed by a hypodermic 27G or 30G needle, there was a distinct linear cut remaining throughout all ocular tissues passed, with no alignment of the cells appearing after the needle was withdrawn (Fig 6a and 6b). The tissues through which the hypodermic needle passed were cut away and a whole tissue column seems to be missing from the needle pathway. Massive subconjunctival and intrascleral hemorrhages were noticed also in few cases. Additionally, erythrocytes, plasmorrhagia and hemorrhagia were noted along the needle track in the ciliary body. The hemorrhages were mostly located within the needle pathway. In cases performed by the Pencan 27G needle, it was shown that due to a push-out effect by the pencil point tip, the cells, especially those within the sclera, appeared to undergo lesser cellular damage (Fig 6c). The collagen fibrils were partially torn apart, the rest of them being preserved by the push-out effect of the Pencan 27G needle; upon removal of the needle, there appeared to be a partial filling-back effect in the needle track as well (Fig 6c). Similar cellular reaction was noticed in the ciliary body pathway of Pencan 27G needle. Interestingly, the incidence of hemorrhages was also lower with this needle type, while a smaller square cut compared to hypodermic 30G needles was located around the needle track and was being observed in all cases.

#### Study on human cadaver eyes

The study results of the entry sites on human cadaver eyes corresponded well to the results obtained on rat eyes. It was shown that the use of hypodermic 30G needle leaves similarly a linear cut of the eye wall tissues, cutting off a tissue column (Fig 6d). There were also single erythrocytes noticed within the needle track. In contrast to the hypodermic needle, Pencan 27G needle presented similarly the push-out effect on cadaver eye sclera as well. The collagen fibrils were partially destroyed, while those that were partially pushed out to the sides, could return back to fill in the needle track in the sclera and the ciliary body (Fig 6e).

### Cultivation of cell aspirates from human cadaver eyes ex vivo

**Fig 7:**
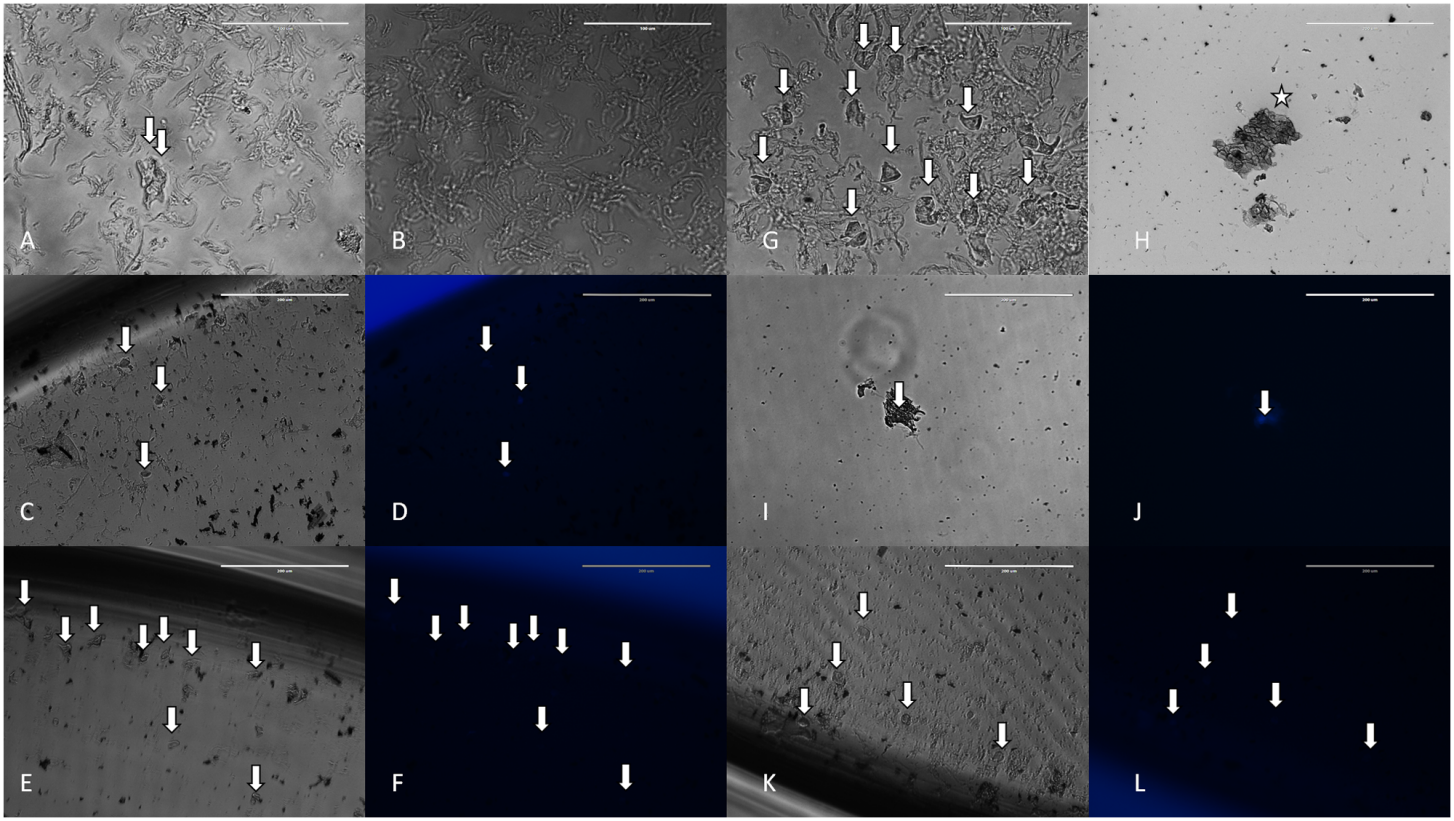
Ex vivo cultivation of cell aspirates from cadaver eyes. Cell conglomerates, single cells, vitreous remnants in hypodermic 30G (a-f) and Pencan 27G (g-l) needle aspirates of human cadaver eyes. Nuclei were stained by Hoechst (d, f, j, l). White arrows point out on cells, while the white star shows a cell aggregate. Scale bar:a,b,g,h = 100μm; c,d,e,f,i,j,k,l = 200μm.

Cells aspirated from the hypodermic 30G and Pencan 27G needles being used on cadaver eyes underwent ex vivo cultivation in cell growth medium accordingly. Significantly less amount of cells was found adherent to the cell culture dish after 24hrs of seeding in case of hypodermic 30G needle (Fig 7a and 7b) compared to the Pencan 27G (Fig 7g and 7h). The adherent or gravitationally immobile cells were mostly of conjunctival cell origin. More single cells and vitreous remnants could be found in the hypodermic 30G needle aspirates (Fig 7a and 7b), while more conjunctival cell conglomerates were observed in the Pencan 27G needle aspirates (Fig 7h). No ciliary body epithelial cells, nor scleral cells could be noticed in the *ex vivo* cultures. After 4 weeks of cultivation, only conjunctival cells could be detected in the culture wells (Fig 7c, 7d, 7e, 7f, 7i, 7j, 7k and 7l).

## Discussion

Despite existence of updated guidelines for the IVI technique, to date, no unique, standard IVI protocol has been established that is widely accepted among all eye surgeons[1,28]. A commonly used IVI technique employs the use of standard hypodermic needle of different gauges[28]. The hypodermic needles, due to the way they are manufactured, have both an outer and inner sharp edge[17]. Assuming similarity to fine needle biopsy needles, during IVI, the hypodermic needles excise a column of the tissue which it passes through. The standard IVI technique uses 27G and 30G hypodermic needles, while we hereby show an alternative way of penetration using Pencan 27G spinal anesthesia needle in both, rat and human cadaver eyes, demonstrating a consequent ocular tissue damage which differs in its extent and is needle-type dependent. During the study of needle tip aspirates, among the most frequently seen cells were conjunctival epithelial-, ciliary body non-pigmented epithelial- and sclerocyte-like cells and vitreous body remnants. Indeed, the use of hypodermic needles with sharp inner edges leads to a tissue column being cut and thus, posing the eminent risk of cellular content being injection and disseminated inside the vitreous body. Although the amount of conjunctival cells, conjunctival cell complexes and ciliary body epithelial cells was higher in aspirates of Pencan 27G needle with pencil-point tip design, it is to note that this needle is not designed specifically for IVI use. On the other hand, the push-out effect applied to the ocular tissue using the 27G Pencan needle facilitated less cellular damage compared to the hypodermic 27G needle. A proof of that was the presence of significantly higher amount of basophilic granulated protein sediment in hypodermic 27G needle aspirates, which indicates damage of the microvasculature, connective and soft tissues. Additionally, the histological study of the needle entry site showed that during penetration, the hypodermic 27G and 30G needles cut off a tissue column, while the Pencan 27G needle pushes aside the cells, with the tissue assuming partial back positioning of the cells within the needle track. The ex vivo cultivation of the cells taken from both hypodermic 30G and Pencan 27G needle tip aspirates further proves that gradual adherence and growth of mainly conjunctival cells within 24hrs and up to 4 weeks occurs, with bigger amount of cells being present in the Pencan 27G needle group, since it is not specific for the purpose of giving IVI.

A report on the incidence of pain and vitreous reflux relative to the needle size during IVI has been provided[29]. It concluded, that use of 33G needles does not reduce the incidence of pain compared to 30G needles. The extent of pain was mostly associated with psychological factors, such as pain expectation, previous IVI experience, as well as individual pain tolerance and pre- and post-IVI aseptic measurements applied to the eye surface. However, the use of thinner needles resulted in less vitreous reflux through the entry site. Similar vitreous reflux studies were carried out independently by De Stefanoet al. and Hubschmanand et al. with similar results being reported[15,30]. In the same study, De Stefano et al. studied the size of scleral hole after IVI evaluating the diameter of injection orifice stained with trypan blue [15]. They noticed that needles with bigger gauge (thinner needles) cause less ocular damage, and that tunneled incisions have higher probability of scleral opening reduction [15]. In our study, the main focus was not to study the vitreous reflux, but to perform histological analysis and comparison of the entry sites using standard hypodermic needles and a needle designed for spinal anesthesia. We show that hypodermic needles cut off the column of all tissues being passed by the needle tip, leaving the tissue defect after the withdrawal, which certainly can be the cause of a vitreous reflux and risk of vitreous contamination via the entry site. In contrast, the entry sites following penetration by spinal anesthesia needle with pencil-point tip showed partial sparing and back positioning of the cells within the needle pathway. In the past, the 33G needle have been shown to require less force to penetrate the sclera compared to 30G needles, but the difference was relatively small. In our study, the same results were observed, meaning that IVIs with hypodermic 27G and Pencan 27G needles necessitate a bigger force[29,31], while in addition, the aspirates taken by the needles with bigger diameter -the hypodermic 27G and Pencan 27G needles, showed a larger amount of cellular content compared to hypodermic 30G needle. It should be kept in mind that Pencan 27G needles are not designed for IVI, thus being much longer and having a big side port. It is the bigger applied force which is hereby assumed to have cause relatively bigger trauma of the eye wall compared to the standard hypodermic IVI needles. It is also suspected that the big diameter of the side port is responsible for collecting larger amount of cells (conjunctival complexes and vitreous strands) during the aspiration. However, the hypodermic 27G needles caused greater damage to the cellular wall of every tissues (vascular, connective, epithelial) within the needle pathway created by the cut, which was proven by the significantly larger amount of granulated proteins. The cutting properties of the hypodermic 27G and 30G needles probably were also responsible for the greater amount of conjunctival complexes including basal membrane compared to Pencan 27G needles.

In a prospective study by de Caro et al.abacterial contamination of the needles used for IVI could be demonstrated even when prophylaxis with antibiotics and povidone iodine was used[14]. The same bacteria (coagulase-negative Staphylococci) were isolated from the needle tips and from the injection site bulbar conjunctiva samples taken from same patients following IVI; it was suggested that there was direct inoculation of ocular surface bacteria into the vitreous body through the injection needles, but the mechanism of bacterial entry into the vitreous remained unknown[14]. We hereby show that ocular tissues including ocular surface cells have been cut by the inner sharp edge of the standard hypodermic needles and become entrapped into the tip’s canal, thus consequently injected into the vitreous with the drug. Additionally, we demonstrate that the configuration of the entry site differs in cases performed with hypodermic and spinal anesthesia needles. The hypodermic needles leave a traceable pathway due to the tissue cut that can be responsible for a vitreous reflux, contrary to the pencil-point needle that pushes out the cells to the side within the pathway, leading to their partial back positioning after the needle withdrawal. Further studies are needed to design new needle tips in order to avoid the inoculation of the cells into the vitreous and support self-sealing properties of the wound after IVIs.

Based on the result of our study we strongly hypothesize that cellular material, that wasreviled after the eye wall penetration using the aspiration technique, gets injected into the vitreous under the real conditions during each IVI in each patient. The risks and the further role of injected cells together with the drug has to be considered. Therefore, there are two possible ways of vitreous contamination during IVI that can be alone or co-existent: 1) intraoperative injection of contaminated cellular cut from the ocular surface, and 2) postoperative contamination of the needle entry site via reflux. The later can happen after the injection in different time intervals. Injected cellular material can be responsible for aseptic, septic or even autoimmune reaction of the vitreous on IVI as well.

The presence of the conjunctival cellular complexes supported by a basal membrane, and the slow but clearly evident growth of cells taken from the aspirates and cultivated in growth medium in vitro, support the hypothesis that injected cells have the ability to grow inside the vitreous body or onto the inner ocular surface, leading to local proliferative reaction.

The Pencan 27G needles have been designed for spinal anesthesia and have a pencil-point atraumatic tip aimed to protect against vascular puncture and nerve damage. The amount of conjunctival epithelial- and ciliary body epithelial- cells, as well as conjunctival complexes and aspirated vitreous remnants was significantly bigger in case of Pencan 27G needle (p = 0.05) compared to hypodermic needles. We assume that it was due to the bigger side size compared to the orifice diameter of the hypodermic needles. It was also noticed that in order to perform a penetration of the sclera with Pencan 27G needle, a two hand assisted IVI and a bigger force were required. A limitation of the study on the cadaver eyes consists in the difference in tissue response to the IVI comparing to the living tissues of the rats’ eyes. The cytological study of the needle tip aspirates in cadaver eyes showed less cellular material, presumably due to the more appropriate size of the eye globe as compared to rats and due to a more specific reaction of cadaver eye tissue to IVI. Nevertheless, the quantity distribution of the cells within the aspirates corresponded in both studies, as well as the results from the study of the entry sites.

In order to study the cellular content of the needles after penetration of the eye wall, we aspirated the vitreous with the aim to get the cells cut by needle edges into the syringe. After IVI, the cellular content is injected into the vitreous cavity, where it probably spreads. We performed no further study on the injected cells into the vitreous, and this might be a limitation of the study. However, the collection of cells from the vitreous cavity would be challenging. Another limitation relates to the cultivation of cells in media *in vitro* but not in the vitreous, because the proliferation of the aspirated cells in the vitreous itself *in vivo* can somehow be different.

Overall, hypodermic 27G needles showed a cut containing bigger amount of sclerocyte-like cells and cellular complexes, as well ascaused a bigger damage to the cells of the eye wall, which was proved by the higher amount of granular proteins. Hypodermic 30G needles showed a cut containing lesser amount of cells and cellular complexes, with amount of granular proteins significantly less compared to hypodermic 27G and Pencan 27G needles. The further use of hypodermic 27G needles for IVI has to be reconsidered.

According to the present study, few possible ways of contamination of the vitreous cavity are assumed: one can be associated with the injection of cellular columncut from the ocular surface which can be contaminated with an infection agent (this can happen intraoperatively); the other can be associated with the insufficient sealing of the entry site via the needle track (this can happen after the injection in different time intervals).

## Conclusions

The IVI technique with the use of subcutaneous or spinal anesthesia needles leads to damage of all eye wall layers on its way (conjunctiva, sclera, ciliary body). The risk of cellular structures’ capture by the needle orifice is high and can lead to injection of autologous, and/or contaminated cellular material into the vitreous cavity. Our data support the hypothesis that sharp circumferential inner edge of standard subcutaneous needles, routinely used for IVI, can cut out a certain amount of cells in rats and cadaver eyes. The diameter of the needle plays a role in the severity of tissue damage and appropriate drug distribution. Additionally, needle tip design determines the configuration of the entry site and possible vitreous reflux respectively. Overall, this study supports that hypodermic 30G needles cause less tissue damage compared to hypodermic27G and Pencan 27G needles. Overall, this study supports that hypodermic 30G needles cause less tissue damage compared to hypodermic27G and Pencan 27G needles. Overall, this study supports that hypodermic 30G needles cause less tissue damage compared to hypodermic27G and Pencan 27G needles. However, the role of the cellular content that has been injected into the vitreous cavity together with the drugs during IVI has to be taken into consideration to better understand the post-injection inflammatory reaction.

## Supporting information

S1 File Supporting data. Data from the amount of granular proteins, conjunctival cell complexes and vitreous body cells in the aspirates taken by different needle types applied to rat eyes, and the amount of conjunctival cell complexes in aspirates taken by hypodermic 30G- and Pencan 27G- needles applied to cadaver eyes.
(XLSX)
